# Joint Trajectories of Performance-Based and Self-Reported Physical Functioning in Older Adults: A 20-Year Longitudinal Study in the Netherlands

**DOI:** 10.1177/08982643241273298

**Published:** 2024-08-21

**Authors:** Dorly J. H. Deeg, Emiel O. Hoogendijk, Natasja M. van Schoor, Laura A. Schaap, Valéria Lima Passos

**Affiliations:** 1 Department of Epidemiology and Data Science, Amsterdam Public Health Research Institute/Ageing and Later Life Program, 1209 Amsterdam University Medical Centers – Vrije Universiteit Amsterdam, Amsterdam, the Netherlands; 2 Faculty of Science, Department of Health Sciences, Amsterdam Public Health Research Institute/Ageing and Later Life program, Amsterdam Movement Sciences, 522567 Vrije Universiteit Amsterdam, Amsterdam, the Netherlands; 3 Maastricht University, Faculty of Health, Medicine and Life Sciences, Department of Methodology and Statistics, CAPHRI Care and Public Health Research Institute, 8863 School of Pharmacy and Biomolecular Sciences, Royal College of Surgeons, Dublin, Ireland

**Keywords:** grip strength, walking speed, disability, group-based multiple-trajectory modeling, disablement process

## Abstract

**Background:**

The well-known disablement process has been conceptualized as a series of transitions between progressive states of functional decline. We studied joint patterns of change within disablement states defined as walking speed, grip strength, and self-reported disability.

**Methods:**

1702 participants aged 65 and over were included from the Longitudinal Aging Study Amsterdam, spanning seven waves over 20 years (1996–2016). Group-based multi-trajectory modeling yielded trajectory clusters (TCs) of different patterns of change, further characterized by baseline sociodemographic characteristics, physical and cognitive health, and survival rate.

**Results:**

Five TCs were identified, distinguished by increasing baseline age. Walking speed and disability showed generally concomitant trajectories. Women had poorer trajectories in grip strength than men, but not in walking speed and disability. Poor physical health distinguished especially the poorest, and cognitive impairment distinguished especially the one-before-poorest from the better TCs.

**Discussion:**

The findings suggest that the disablement states are not generally distinct or sequential.

## Introduction

Functional ability is widely recognized as central to aging well. Regardless of its definition, ample evidence shows that decline in functional ability is associated with adverse physical and mental health outcomes and mortality (Cooper et al., 2010, 2011, 2014). Functional ability is, in fact, an umbrella term for several aspects of physical functioning, including mobility, strength, and disability in daily activities and self-care. A widely used model linking the components that may constitute functional ability is the Disablement Process Model (DPM) (Verbrugge & Jette, 1994). In this conceptual model, four states are distinguished, between which transitions occur in the course of aging: disease processes, impairments in bodily organs, limitations in basic actions, and disability in activities. Since the DPM was launched, variations on the model have been proposed. A recent extension of the DPM conceives of the limitations state as two distinct sub-states, that is, limitations in one body system and limitations in multiple body systems (Beaudart et al., 2019).

The DPM stipulates that disability is the gap between a person’s capacity and the requirements of the environment in which this person lives. Accordingly, intra-individual as well as extra-individual factors are proposed to affect the transition rate from one state to another. Transitions between successive states and several of their determinants have been supported by empirical evidence (Guralnik et al., 2000; Heiland et al., 2016; Lawrence & Jette, 1996; Peek et al., 2003; Rouquette et al., 2015; van Gool et al., 2005). These empirical studies assess the predictive ability of one state at baseline, for example, impairment, for another state, for example, functional limitations a few years later, or they examine the mediating role of functional limitations in the association between impairments and disability over time.

Despite its much needed clarification of the various states of disablement, a criticism of the DPM is that the ‘states’ described in it are not mutually exclusive. In cross-sectional studies, for example, overlap between impairments, functional limitations, and disability ranged between 18 and 33% (Deeg & Puts, 2007; Fried et al., 2004; Zamudio-Rodriguez et al., 2020). More dynamically phrased, impairments may not be pervasive for limitations in basic actions to emerge, and basic actions may not be fully limited before the onset of some extent of disability. This implies that over time, intra-individual change in the components of disablement may be simultaneous rather than consecutive. Even more likely, the rate of change in DPM components may differ within individuals and limitations in some basic actions may lead to disability, and in some others, may not (Kempen et al., 1998). The pattern of possible trajectories may also depend on factors that in the DPM are proposed to affect transitions between states. Thus, the disablement process is likely to manifest itself in multiple patterns of change over time, and these patterns may be characterized by a range of factors.

Dynamic approaches to disablement have emerged relatively recently, thanks to the advancement of statistical modeling tools (Verbrugge, 2016). These approaches involve examining intra-individual change in functioning over time, distinguishing different trajectories such as ‘stable high functioning’, ‘high and declining’, or ‘low and declining’, using latent class growth or group-based trajectory models (Hoekstra et al., 2020; Liang et al., 2010; Martin et al., 2015; Rooth et al., 2016; Wolinsky et al., 2011; Yu et al., 2015; Zimmer et al., 2012). These studies generally focus on one component of disablement, and thus do not provide insight into similarity or dissimilarity to trajectories in other components or interrelations between components. In one study, the overlap between separately determined trajectories of four functional limitations (tandem stand, gait speed, chair stand, and handgrip strength) was shown to range from 12% in younger-old men to 6% in younger-old women (Hoekstra et al., 2020). Although the latter study substantiates the disparity of trajectories over time, it provides a rather simple representation of this disparity as ‘overlap’ versus ‘no overlap’. Very few studies so far identified joint trajectories of disablement states. Pan and colleagues (2021) modeled joint trajectories of disability in activities of daily living and in instrumental activities of daily living, showing three generally corresponding trajectories, with less favorable trajectories in IADL than in ADL. This study, however, focuses on disability and disregards other states in the disablement process.

The starting point for the current study is the expectation that the combination of trajectories of several components of functional ability may be most informative regarding the dynamics of the disablement process. As we consider the later states of the disablement process as most critical to functional decline, we focus on changes in muscle strength to indicate limitations in one body system, in walk ability to indicate limitations in multiple body systems, and in disability. The earliest state of disablement, the presence of diseases, is included as baseline indicator (Martin et al., 2015). From the empirical insights obtained, preventative measures can be derived that focus on the functional ability component that declines faster than others, or, if a person already is declining in one component, focusing on maintaining functional ability in the component that has been stable so far (Gore et al., 2018).

The current study, therefore, aims to identify joint patterns of change over 20 years in three components of functional ability. To this end, longitudinal data will be analyzed using group-based multi-trajectory modeling (Nagin et al., 2024). Group-based trajectory modeling identifies subgroups of individuals following similar trajectories over time; that is, it identifies the number and shapes of the most common trajectories in a single measure of functional ability (e.g., Martin et al., 2015). The multivariate version of group-based trajectory modeling furthermore identifies clusters of trajectories representing distinct patterns of joint development. This approach is often used to model dynamic processes of several inter-related, co-developing measures. In our study, we aim to determine clusters of trajectories representing distinct patterns of joint development of muscle strength, walk ability and disability.

Our basic hypothesis corresponds with the extended DPM and involves time-lagged changes, in particular, change in muscle strength precedes change in walk ability, which in turn precedes disability. This would be apparent from a cluster of trajectories in which muscle strength declines substantially earlier in the study period, whereas walk ability declines only later on, and disability starts increasing even later on in the study period.

Alternatively, we consider that the DPM components likely co-develop in a variety of patterns. This would amount to the existence of multiple subgroups with unique experiences of disablement, represented by clusters of trajectories which are distinguished by their unique temporal patterns of co-development. Furthermore, we hypothesize that these subgroups can be distinguished based on the characteristics age, sex, education, physical and cognitive health, and survival. As such, our study provides building blocks for further conceptualization of different experiences of disablement.

## Methods

### Data and Sample

We used data from the Longitudinal Aging Study Amsterdam (LASA). The study was initiated in 1992–1993, with a nationally representative sample of 3107 participants aged 55–85 years living in 11 municipalities in the Netherlands. Measurement waves included face-to-face interviews at home with follow-up intervals of 3 or 4 years (Hoogendijk et al., 2020). For this study, we selected 1759 individuals aged 65 years and over who participated in the first wave in which grip strength was measured, that is, 1995–1996 (T1). Follow-up waves took place in 1998–1999 (T2, *N* = 1362), 2001–2002 (T3, *N* = 1007), 2005–2006 (T4, *N* = 646), 2008–2009 (T5, *N* = 487), 2011–2012 (T6, *N* = 318), and 2015–2016 (T7, *N* = 169). We included participants who had valid data on at least any two of the three components of functional ability for at least the first wave, in order to allow initially already low-functioning participants to contribute information. This yielded a study sample of *N* = 1702. For these participants, the total number of observations was 4817 for grip strength, 4953 for walk ability, and 5378 for disability, which amounts to availability of data for on average three waves. Attrition was associated with higher baseline age and poorer baseline physical and mental health, but not with sex and level of education (Supplement eTable 3a).

### Measures

#### Functional Ability

The DPM state of functional limitations was measured as handgrip strength and walk ability, defined as time needed to walk a short distance. These measures are considered to be distinct, as the former involves one body system, that is, muscle, and the latter involves multiple body systems including neurological and cardiovascular systems (Beaudart et al., 2019). Both measures are widely used to predict disability outcomes (Cooper et al., 2010, 2011) and have been shown to complement one another in the prediction of mortality and functional decline (Nofuji et al., 2016; Tange et al., 2022). Handgrip strength was measured with a handheld dynamometer (Takei TKK 5001, Takei Scientific Instruments Co. Ltd., Tokyo, Japan). The measurement was performed with two repeated measurement attempts per hand, while participants were in standing position. The maximum value out of four attempts (in kg) was used in the analyses. For walk ability, participants were asked to walk 3 m, turn around and walk back, as quickly as possible, resulting in a 6 m walk. Because of the skewness of this measure, its natural log was used in the analyses.

The DPM state of disability was self-reported based on questions about difficulty or needing help in performing six activities, including walking up and down stairs; using own or public transportation; cutting own toenails; dressing and undressing; sitting down in and standing up from a chair; and walking outside for 5 minutes without stopping. The response options were: 0. Yes, without difficulty; 1. Yes, with some difficulty; 2. Yes, with much difficulty; 3. Only with help; 4. No, I cannot. The summed score ranged from 0 to 24, with higher scores reflecting more and/or more severe disability. Internal consistency was high, with Cronbach’s alpha’s ranging from 0.84 to 0.88 across waves.

#### Survival

Vital status was ascertained through municipal registries. It was complete but for 4 cases.

#### Covariates

The covariates were measured at baseline (1995–1996). Age and sex were derived from the population registry. Educational level was self-reported as the highest level of education completed, and converted to number of years of education. Physical health was assessed as the number of self-reported chronic diseases, specifically, chronic non-specific lung disease, cardiac disease, peripheral arterial disease, diabetes mellitus, stroke, arthritis, cancer, and a maximum of 2 other diseases, and ranged from 0 to 8. Cognitive health was assessed using the Mini-Mental State Examination (MMSE). Scores range from 0–30, with higher scores indicating better cognitive functioning (Folstein et al., 1975).

### Analytic Strategy

Joint patterns of change in the three functional ability measures were estimated using group-based multi-trajectory modeling (Klijn et al., 2017; Nagin et al., 2018). The procedure involved several steps, accounting for variation in both level and shape, the details of which are provided in Supplement 1. We denote the resulting joint patterns of change as trajectory clusters (TCs).

The TCs obtained were then characterized by the sociodemographic and health covariates and survival rate. In unadjusted analyses, the TCs were compared using chi-square tests for categorical covariates and F-tests for continuous covariates. In adjusted models, the associations of the covariates with each TC was assessed by odds ratio’s (ORs) and 95% confidence intervals (95% CIs) from multinomial regression analysis, taking the highest-functioning TC as the reference. To account for non-linear associations, all continuous variables were categorized.

The analyses were conducted using *proc traj* in SAS (v. 9.4 SAS Institute, Cary, NC, USA), class-enumeration was assisted by the Fit-criteria Assessment Plot (F-CAP) in RStudio (v. 3.6.3; RStudio, PBC, Boston, MA, USA), and the descriptive characterization and multinomial analysis were performed in SPSS (v. 28, IBM Corp, Armonk, NY, USA).

## Results

### Sample Description

The average age of our study sample is 75.8 (sd 6.7) years at baseline and 89.3 (sd 3.8) at the last wave (T7); 52.9% are women; and the average number of years of education is 8.8 (sd 3.3) years, indicating elementary schooling plus a few years of general or vocational schooling.

### Determining Joint Trajectory Clusters

**Figure 1.**
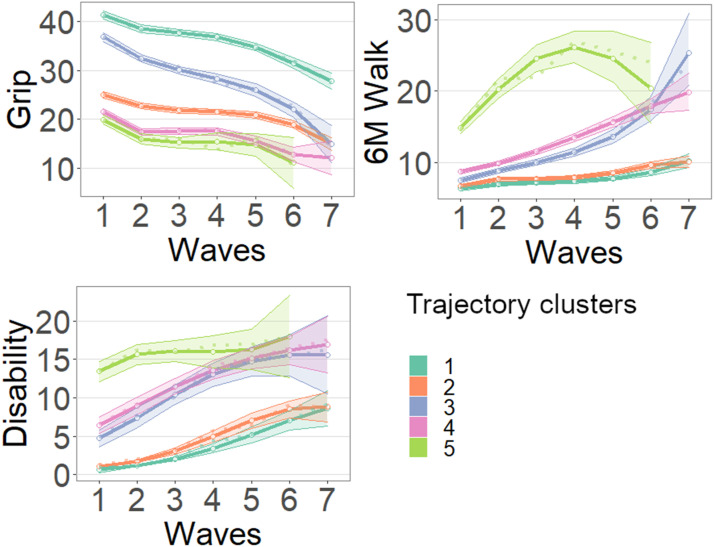
Multivariate trajectories of physical functioning: grip strength (kg), walk ability (6 m walk in seconds), and disability (sum of scores: 0–24). Baseline prevalence: 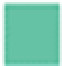
TC1 – High-functioning (16.4%), 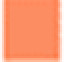
TC2 – High-functioning, below-average grip strength (24.0%), 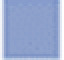
TC3 – Functional decline (16.3%), 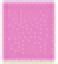
TC4 – Functional decline, poor grip strength (28.5%) and 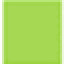
TC5 – Low-functioning (14.8%). Dotted lines: observed averages; Full-lines: predicted averages with 95% confidence bands. For the underlying values, see Supplement eTables 2a–c.

A number of five TCs showed the best model fit, as indicated by the fit criteria (Supplement eTable 1). Figure 1(a)–(c) show the five trajectories for each functional ability measure. Trajectories belonging to the same cluster have the same color. The TCs are distinguished based on intercept and rate of change and numbered as 1 to 5 from most to least favorable.

TC1 (dark green, estimated prevalence 16.4%): ‘High-functioning’. It represents the best scores on all three measures of functional ability throughout the study period. It is characterized by the best intercept and very little deterioration over time, with a slight acceleration in the second half of the study period.

TC2 (orange, 24.0%): ‘High-functioning, below-average grip strength’. It represents scores almost as good as TC1 for the 6m walk and for disability, but relatively poor performance for grip strength. The latter has a relatively poor intercept with only little deterioration throughout the study period.

TC3 (violet, 16.3%): ‘Functional decline’. It is less robust than TCs 1 and 2 for the 6 m walk and for disability, with somewhat poorer intercepts. The intercept of grip strength is better than in TC2 and only slightly worse than in TC1. Declines in all three measures are relatively steep, for grip strength and the 6 m walk particularly during the second half of the study period, whereas the decline in disability flattens during this period. Grip strength shows the greatest decline compared to the other TCs.

TC4 (pink, 28.5%): ‘Functional decline, poor grip strength’. For the 6 m walk and disability, it largely coincides with TC3, with slightly poorer intercepts but similar slopes. Grip strength runs parallel to TC2, with a slightly poorer intercept.

TC5 (bright green, 14.8%): ‘Low-functioning’. The intercepts are most unfavorable, in particular for the 6 m walk and disability. For the 6 m walk, the decline is steepest in the first half of the study period. For grip strength and disability, however, very little decline is observed, but instead, prolonged low functioning. The widening confidence intervals indicate relatively large attrition in the course of the study period (see Supplement eTable 3b).

### Further Characterization of the Trajectory Clusters

Baseline age increases from 71 years in TC1 to 81 years in TC5. TC1 and TC3 are dominated by males (>90%), whereas females are more often categorized in TC2, TC4, and TC5 (69%–74%) (Table 1). The number of years of education declines monotonically from 10 years in TC1 to 8 years in TC5. The number of chronic diseases at baseline increases from 1.1 in TC1 to 2.5 in TC5, with sharp increases between TC2 and TC3 and between TC4 and TC5. Baseline cognitive health decreases from MMSE-score 27.6 in TC1 to 24.8 in TC5, with clear decreases after TC3. The percent survivors across the 20-year study period drops from almost one-third (TC1, TC2) to almost zero for TC5. Correspondingly, among the deceased, the time to death is longest for TC1 and TC2 at about 11 years, drops to about 9 years for TC3 and TC4, and is only 6 years for TC5. All covariates differ significantly across the TCs.

**Table 1.** Socio-Demographic and Health Characteristics of the Five Trajectory Clusters.

	Trajectory Cluster (TC) Number and Name^a^						
	TC1: High-Functioning	TC2: High-Functioning, Below-Average Grip Strength	TC3: Functional Decline	TC4: Functional Decline, Poor Grip Strength	TC5: Low-Functioning	Total	*p*-Value^a^
	*N* = 258	*N* = 394	*N* = 245	*N* = 506	*N* = 299	*N* = 1702	
Age (M, se)	71.0 (0.3)^I^	72.7 (0.3)^II^	75.6 (0.4)^III^	77.6 (0.3)^IV^	81.3 (0.3)^V^	75.8 (0.16)	<.001
Sex (% females, se)	4.7 (0.2)^I^	72.6 (0.4)^II^	8.2 (0.3)^I^	74.5 (0.4)^II^	68.9 (0.5)^II^	52.9 (1.21)	<.001
Years of education^b^ (M, se)	10.2 (0.2)^I^	9.0 (0.2)^II^	9.3 (0.2)^II^	8.2 (0.1)^III^	8.0 (0.2)^III^	8.8 (0.08)	<.001
Number of chronic diseases^b^ (M, se)	1.1 (0.1)^I^	1.2 (0.1)^I^	1.9 (0.1)^II^	1.9 (0.1)^II^	2.5 (0.1)^III^	1.8 (0.03)	<.001
MMSE score^c^ (M, se)	27.6 (0.1)^I^	27.5 (0.1)^I^	27.0 (0.1)^I^	26.0 (0.2)^II^	24.8 (0.2)^III^	26.5 (0.08)	<.001
Survived up to last wave^d^ (%, se)	31.8 (0.5)^I^	32.1 (0.5)^I^	7.8 (0.3)^II^	9.9 (0.3)^II^	1.7 (0.1)^III^	16.6 (0.9)	<.001
Deceased: Survival in years^d^ (M, se)	10.7 (0.4)^I^	11.2 (0.3)^I^	9.5 (0.3)^II^	9.0 (0.2)^II^	6.3 (0.3)^III^	9.1 (0.14)	<.001

*Note.* M = mean; se = standard error.

^a^The *p*-value provides an overall significance of differences; significantly different TCs are indicated by different Roman numbers in superscripts.

^b^2 missing data, both in TC5.

^c^5 missing data: 2 in TC2, 1 in TC4, 2 in TC5.

^d^4 missing data: 1 in TC2, 3 in TC4.

**Figure 2.**
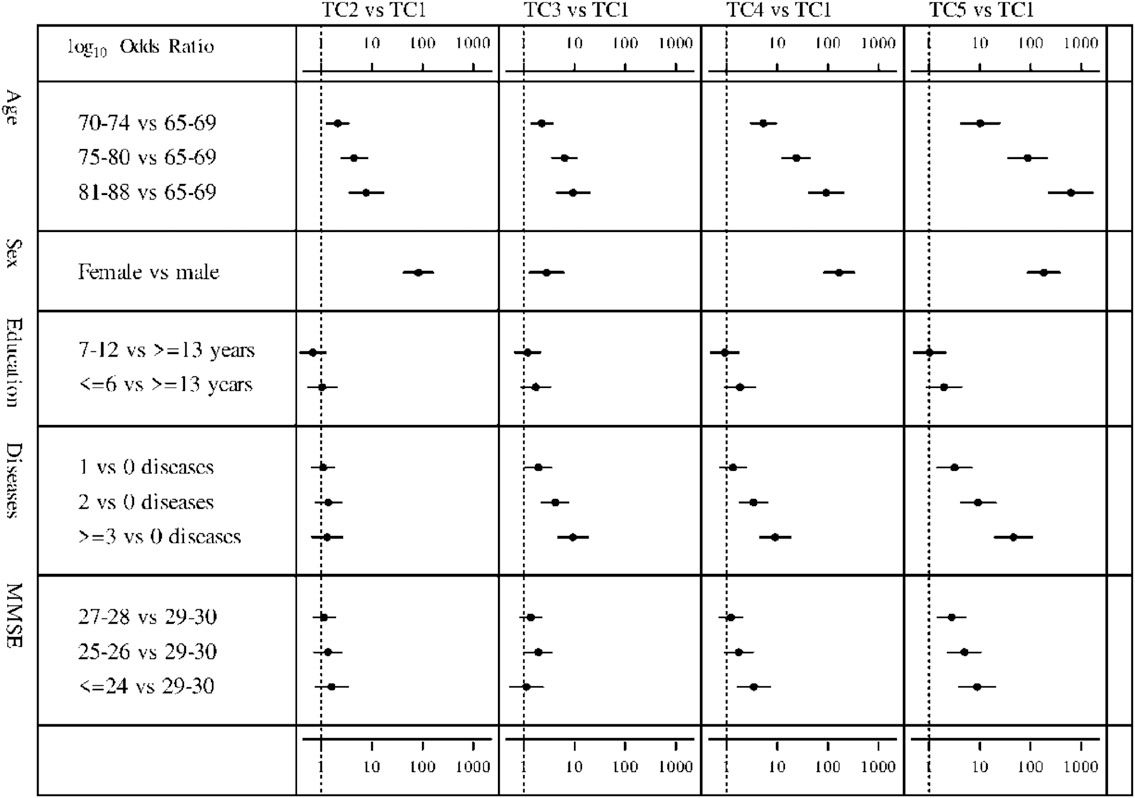
Comparison of trajectory clusters (TCs) 2–5 with the reference cluster TC1 regarding the sociodemographic and health covariates: forest plots of the results from multinomial regression analysis. Odds ratios (ORs) and confidence intervals are presented on a ^10^logarithm scale. The dotted line indicates OR = 1. For the original OR values, see Supplement eTable 4.

In the full multinomial regression models, all observed associations of covariates with TCs remain significant except for years of education (Figure 2) (Supplement eTable 4). The two models, first including age and sex (model 1) and then adding the other covariates (model 2), yield explanatory values (Nagelkerke pseudo-R^2^) of 55.7% and 63.8%, respectively.

Based on these results, a full description of the five trajectory clusters is given in Box 1.

**Box 1.** Full Characterization of the Five Trajectory Clusters.

Trajectory Cluster Number, Name, and Estimated Prevalence	Characterization
TC1 – High-functioning (16.4%)	Longer lived, youngest old, predominantly men, well-functioning with only slight declines, despite the presence of one chronic disease on average
TC2 – High-functioning, below-average grip strength (24.0%)	Longer lived, relatively young, predominantly women, well-functioning with slight declines in walk ability and disability, but below-average grip strength, with one chronic disease on average
TC3 – Functional decline (16.3%)	Shorter lived, relatively young, predominantly men, declines in all three measures, with two chronic diseases (multimorbidity)
TC4 – Functional decline, poor grip strength (28.5%)	Shorter lived, relatively old, predominantly women, declines in walk ability and disability, with all along poor grip strength, multimorbidity, and poor cognitive health
TC5 – Low-functioning (14.8%)	Shortest lived, oldest-old, predominantly women, starting at poor levels in all three measures with decline in walk ability and poor levels in grip strength and disability, more multimorbidity, and poor cognitive health

## Discussion

Examining joint patterns of change in three measures of functional ability, we found five distinct trajectory clusters (TCs), highlighting the heterogeneity and the dynamic nature of the trajectories within ‘states’ of the disablement process. Based on their intercepts and rates of deterioration alone, the TCs showed a rank ordering from highest to poorest functioning. However, there were deviations within the rank order. For example, while we observed mostly similar patterns of intercepts and changes in walk ability and disability, the intercepts of grip strength showed a different pattern. For a proper interpretation of the TCs, therefore, we turn to the characterization using the covariates.

Among the sociodemographic and health characteristics, baseline age and sex were the most distinctive in all TCs. Average baseline age increased gradually from TC1 (‘High-functioning’) to TC5 (‘Low-functioning’), corresponding to the gradual increase in intercepts for the 6 m walk and disability.

Regarding sex, men dominated TC1 and TC3 (‘Functional decline’), while women dominated the other TCs. Whereas TC1 was the most robust, a distinctive characteristic of TC3 was the relatively steep decline in grip strength and walk ability over the later waves, but not in disability. TC2 (‘High-functioning, below-average grip strength’) and TC4 (‘Functional decline, poor grip strength’) particularly distinguished themselves by a poor grip strength, whereas walk ability and disability trajectories overlapped with TC1 and TC3, respectively. A poorer grip strength of women in comparison to men has been shown across many countries (Crimmins et al., 2019). Poor grip strength has been shown to increase mortality risk independent of other functional ability measures, particularly in women (Eekhoff et al., 2019). Thus, one would expect that survival in TC2 would be poorer than in TC1, and again in TC4 poorer than in TC3. However, our findings show that survival is similar in TC2 to TC1 and in TC4 to TC3. This can be understood considering that men dominate TC1 and TC3 and male survival is generally lower than female survival, even when confronted with similar health problems (Case & Paxson, 2005).

Surprisingly, education showed no added value in characterizing the TCs; in the multinomial regression model, education only marginally improved the model fit after age and sex were accounted for (Supplement eTable 5). This contrasts with previous rather consistent evidence that poorer levels of education are associated with less favorable trajectories (Kok et al., 2016; Tange et al., 2022; Wickrama et al., 2013; Zimmer et al., 2014). Educational attainment has greatly increased in more recent cohorts of Dutch older people, with older men still having attained a higher educational level than older women. As baseline age may represent both age and cohort, the variation in educational attainment across cohorts may be captured largely by baseline age. Likewise, same-age educational differences may be captured by sex.

Physical health did not distinguish TC2 from TC1, but was distinctive for TC3 and poorer TCs, particularly when comparing TC5 with TC1. Recalling that TC3 was dominated by men, the steep decline in grip strength and walk ability seen in TC3 in the latter part of the study period may indicate that the association of multimorbidity with decline in physical performance is stronger in men than in women. This interpretation is supported by a recent study on grip strength (Svinøy et al., 2023). However, men are known to have greater mortality than women, given a certain level of morbidity (Case & Paxson, 2005; Crimmins et al., 2019). Correspondingly, the share of men contributing to TC3 substantially declined. Thus, the greater deterioration in the second half of the study period may also be a reflection of the greater share of women contributing.

Poor physical health, as indicated by multimorbidity, increased across the TCs, particularly distinguishing TC5 from TC1-4. TC5 was characterized by sharply decreasing walk ability and high, stable disability as well as low, stable grip strength. These findings underline the evidence of a strong association of multimorbidity with disability (Ryan et al., 2015). This suggests that walk ability should be monitored not only in older people before they become disabled, but also in people with multimorbidity and disability, so that measures can be taken to prevent further functional decline.

Cognitive health did not distinguish TC1-TC3, but was distinctive for TC4 and even highly distinctive for TC5. TC4 was characterized by a stable low grip strength and an MMSE-score of 26.0, a score often used to identify individuals at risk of mild cognitive impairment. A meta-analysis of nine longitudinal studies showed a moderate association of rates of change in grip strength and cognitive functioning (Zammit et al., 2021). The authors attribute this association to brain aging, that is, brain atrophy and white matter hyperintensity accumulation, of which muscle strength is indicative. However, this association is thought to emerge only after a certain threshold of atrophy. A study that modeled trajectory clusters of both physical and cognitive functioning found an association only in the low-functioning cluster (Robitaille et al., 2018). Our own findings support this evidence, as the relatively favorable TC2 is also characterized by a stable low grip strength, but by good cognitive functioning at baseline (MMSE-score was the same as in TC1).

We now turn to the extent to which our findings reflect the Disablement Process Model. According to our basic hypothesis of time-lagged change, we should be able to distinguish trajectory clusters which show earlier decline in grip strength and later decline in walk ability and still later decline in disability. This hypothesis could be further specified as resulting in at least three TCs. In one TC, the muscle strength trajectory would decline from early on during the study period, from a high intercept. In a second TC, muscle strength would start from a lower intercept, indicating that decline has already happened before the study period. In these TCs, the walk ability and disability trajectories would start at a good intercept and then decline later on in the study period, following an upwardly curved trajectory. A third TC would then show declines in both muscle strength and walk ability earlier in the study period, while disability starts increasing only later in the study period, again following an upwardly curved trajectory.

We generally found parallel developments of walk ability and disability. In the middle trajectory (TC3), however, from wave 5 onwards, the decline in both grip strength and walk ability accelerated, whereas the increase in disability flattened. In this TC, disability started already at a relatively poor level; in fact, it followed the same course as in the next poorest trajectory (TC4). TC3 clearly speaks against disability increase following declines in grip strength and walk ability. Furthermore, in the poorest trajectory cluster (TC5), disability remained relatively stable at a very poor level, whereas during the first four waves walk ability declined from an intermediate level. These observations would speak to a precedence of increase in disability, followed by a decline in walk ability and, possibly, grip strength. Grip strength showed a contrasting development with both walk ability and disability in TC4, where it started at a relatively poor level and showed little further decline, while both walk ability and disability showed substantial deterioration. This observation may support the notion that decline in grip strength does precede disability. In sum, however, no clear sequence of declines emerges in the performance of basic actions followed by increases in disability.

Instead of viewing the disablement process as time-lagged declines in successive components of disablement, the trajectory clusters identified in our study suggest disablement as composed of simultaneous changes in multiple aspects of functional ability. The five subgroups with unique joint patterns of change and their covariate characteristics that emerged from our study may represent different experiences of disablement. It is good to emphasize that the evidence from our study is preliminary and should be replicated in future research. Such research may use different indicators of disablement, different lengths of follow up, and – ideally – shorter time intervals between measurements. The time interval in our study is rather long and may preclude the identification of time-lagged changes in the functional ability measures taking place within this time interval – and thus preclude the DPM as one of the possible experiences of disablement.

The strength of our study is its multi-trajectory approach, which provided insight into heterogeneity in co-occurring trajectories in three functional ability measures. Moreover, the covariates showed association patterns that were unique to each of the five trajectory clusters. A limitation of our study may be that we included a parsimonious set of covariates. Particularly, as the development of disability is contingent on the demands of the environment, inclusion of indicators of the environment in which activities are carried out and the use of assistive technology would have provided additional insight.

Another strength of our study is that we included as many study participants as we deemed warranted, thus allowing initially already low-functioning participants to contribute information. In contrast, most trajectory studies, but not all (Martin et al., 2015; Zimmer et al., 2014), included participants who provided data on at least two measurement waves, implying that the initial sample is relatively high-functioning (Hoekstra et al., 2020; Seves et al., 2021; Ward et al., 2016; Wolinsky et al., 2011; Zimmer et al., 2012). This likely reduces the observed heterogeneity of trajectories and overstates the proportion of participants in the high-functioning trajectories. Whereas typically, these studies result in a majority of participants in a high-functioning trajectory, in our study the next-to-lowest-functioning trajectory cluster is the largest. A comparison of participants who provided data at a minimum of two waves with those who did not, but did fulfill our more liberal criteria, shows that indeed the latter were older and had poorer physical and mental health at baseline (Supplement eTable 6). Arguably, inclusion of these participants increases the veracity of our findings and decreases the ‘optimistic’ bias attached to their exclusion.

On the downside, our ‘liberal’ inclusion criteria imply that our parameter estimates in the poorer trajectory clusters may be less precise, as they may be based on fewer measurement waves. This is particularly true for the poorest trajectory cluster, in which average survival amounted to only six years and which was based on only two waves on average (Table 1 and Supplement eTable 3b). This imprecision may be considered the price to be paid for inclusion of initially low-functioning participants. Also, attrition throughout the study was higher among older and less healthy participants. This introduces inevitable bias by underestimating the deterioration in the TCs that are most affected by poor initial health. However, had we included only participants who provided data at two or more valid waves, as was done in most other studies, this bias would have been larger.

Particularly in TC5, the confidence limits of the trajectories grew wider apart in the course of the study period, and the estimated trajectory of walk ability seemed to improve. This is likely caused by the comparatively better functioning of some participants in the small number of survivors later on in TC5. Although the confidence intervals in the other TCs were smaller, they highlight remaining variability within each trajectory.

In conclusion, using three measures of functional ability corresponding to successive states of the DPM, we did not find evidence in support of one clear sequential disablement process. Instead, we found evidence on five distinct trajectory clusters showing simultaneous changes in functional ability measures. Our findings suggest different experiences of disablement, based on initial level, rate and shape of change in functional ability, as well as baseline characteristics of each cluster. Our study provides a useful definition of each of these experiences, so that they can be recognized in practice.

## Supplemental Material

Supplemental Material - Joint Trajectories of Performance-Based and Self-Reported Physical Functioning in Older Adults: A 20-Year Longitudinal Study in the Netherlands Supplemental Material for Joint Trajectories of Performance-Based and Self-Reported Physical Functioning in Older Adults: A 20-Year Longitudinal Study in the Netherlands by Dorly J. H. Deeg, Emiel O. Hoogendijk, Natasja M. van Schoor, Laura A. Schaap, and Valéria Lima Passos in Journal of Aging and Health
