# Supplementary material for: Joint Trajectories of Performance-Based and Self-Reported Physical Functioning in Older Adults: A 20-Year Longitudinal Study in the Netherlands
Source: J Aging Health. 2024 Aug 21;37(9):507–16. doi: 10.1177/08982643241273298 (PMC12405651; doi:10.1177/08982643241273298)
Supplement: Supplemental Material - Joint Trajectories of Performance-Based and Self-Reported Physical Functioning in Older Adults: A 20-Year Longitudinal Study in the Netherlands [file sj-pdf-1-jah-10.1177_08982643241273298.pdf]

‘Joint trajectories of performance-based and self-reported physical functioning in older adults: A 20-year longitudinal study in the Netherlands’

Supplementary material

In this supplementary material, readers find details on

1. The multivariate Group-Based Trajectory Model (GBTM) fitting and selection based on the Fit Criteria Assessment Plot (FCAP) with brief explanantion.
2. Four tables (S3-S6) showing results from additional analyses in support of issues raised in the Discussion section.

Supplement 1: Group-Based Trajectory Model (GBTM)

GBTM clusters individuals sharing similar patterns of stability and change of outcome(s) over time (latent trajectories). The advantage of the multivariate version of GBTM is that, besides identifying individual trajectories of each outcome separately, it also describes their (distinct) patterns of joint evolution. We denote these patterns by the term ’trajectory clusters’.

Model fitting

First, the number of latent trajectory clusters was established (‘class-enumeration’) using a supportive R-code that compiles fit-indices for several models with different numbers of latent clusters (Fit-criteria Assessment Plots[1] – F-CAP – for details see below). For this purpose, models with increasing numbers of clusters were run (1 to 9), all with quartic polynomials. After class-enumeration, the non-significant high-order polynomials were pruned (p>=0.05; p-values higher that 0.05 were occasiaonally accepted, once deletion of the higher order led to a substantial deterioration of BIC).
Longitudinal data without a natural starting point (e.g., birth) can be characterized by substantial onset variability. If this happens to be the dominating data feature, mixture models, of which GBTM is a case, may underperform in their shape-detecting capabilities. As a result, data partitioning will be dominated by level, instead of shape, yielding flat, sparse, parallel trajectories, and process heterogeneity in the longitudinal data may go undetected.

- *Ad hoc strategy to improve GBTM’s shape detectability:*

When level variability appears to be the dominating data feature, Heggeseth et al.[2] suggested removing level by subtracting the within-individual mean score from all within-individual observations. Our *ad hoc* strategy to circumvent the problem of sparse data partitioning involved two steps. Similarly to Heggeseth at al., as the first step, we determined the longitudinal shape of an outcome variable $\boldsymbol{Y}$ by centering the scores. The vector of subject specific scores $\boldsymbol{Y_i}$ were centered, i.e. the scores were subtracted from the individual’s average over the time points ($\boldsymbol{\bar{y}_i = t_i^{-1} \sum_{t=1}^{t_i} y_i}$ **),** previous to model fitting. Let $\boldsymbol{A_i = I_{t_i} - t_i^{-1} 1_{t_i} 1_{t_i}^T}$ be the centering matrix. The vector of centered scores for subject *i* were obtained by $\boldsymbol{C_i = A_i Y_i}$. We centered scores for the 6m Walk and Grip strength. As the second step, different from Heggeseth et al., we then coupled centered values back to the original ones by using the multivariate version of GBTM. Thus, for these outcomes original and centered scores were included in the multivariate GBTM (for both, the Censored Normal distribution was applied as link function), together with the disability scores. We included both sets of scores, so that clustering could capitalize on both level and shape. This strategy improved GBTM’s shape-modeling in the presence of high onset variation.

Model fitting proceeded as previously described.

Fit-Criteria Assessment Plots (FCAP) for class-enumeration

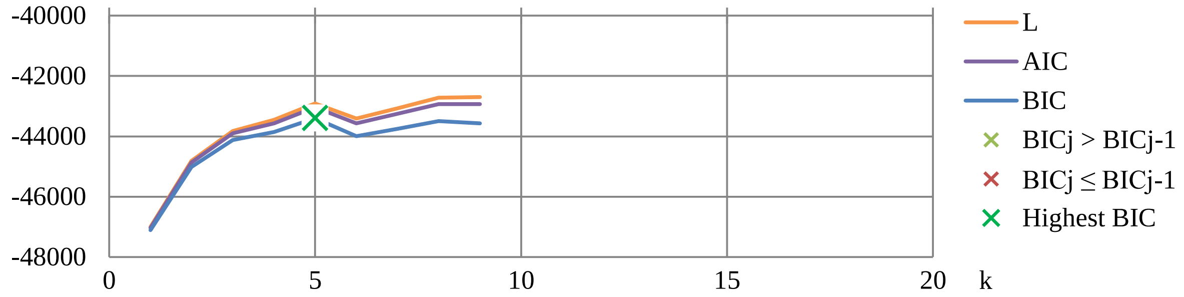

eFigure 1. Akaike's information criterion (AIC), Bayesian information criterion (BIC) and Likelihood

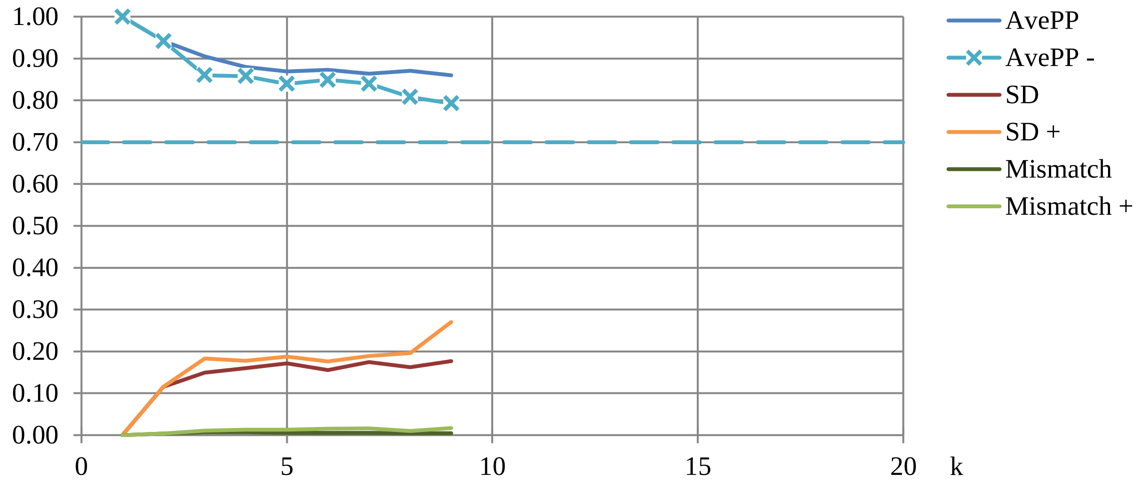

eFigure 2. Average Posterior probability (of assignment), mismatch (between assigned and estimated class probabilities) and SD (of cluster membership probabilities)

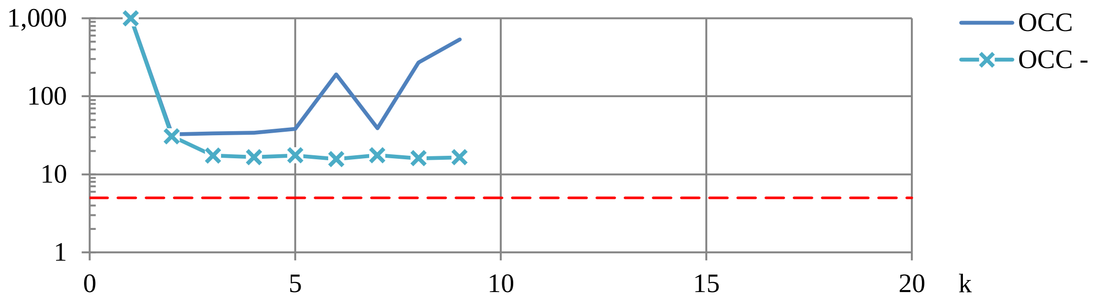

eFigure 3. Odds of correct classification (OCC)

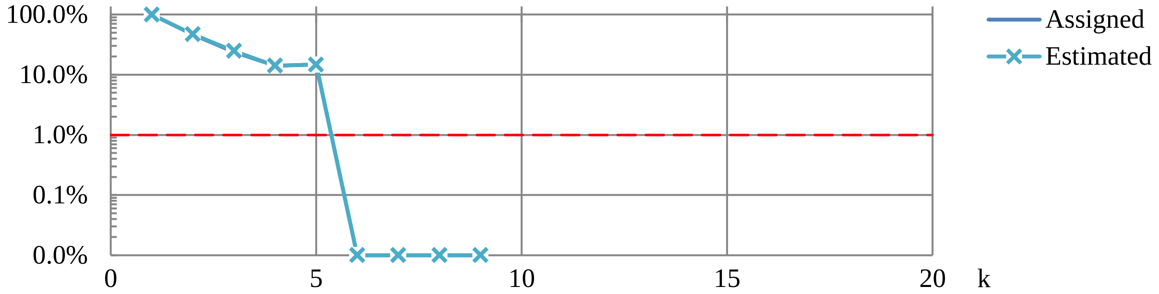

eFigure 4. Percentage of individuals belonging to the smallest cluster. Note that the assigned and estimated lines fully overlap, so that the line 'Assigned' is invisible

eTable 1. Coefficients of fit for 3, 4, 5, and 6 trajectory clusters. The metrics for the final selection and k=5 are highlighted

	BIC	AIC	Likelihood	APP
3 trajectory clusters	-44,118.7	-43,900.4	-43,818.4	0.860
4 trajectory clusters	-43,851.2	-43,563.8	-43,455.8	0.858
5 trajectory clusters	-43,395.6	-43,038.9	-42,904.9	0.839
6 trajectory clusters	-43,990.1	-43,564.2	-43,404.2	0.849

Notes: BIC=Bayesian Information Criterion; AIC=Akaike Information Criterion; APP=Average Posterior Probability.
The BIC, AIC, and Likelihood show a clear maximum at 5 trajectory clusters. The APP is less discriminatory, but remains above 0.80 for all numbers of trajectory clusters explored. In addition, for k>5 the sizes of the trajectory clusters become too small to be further considered. (For all fit parameters from the Fit-Criteria Assessment Plot, see Supplement 1).

Brief explanation of the FCAP:

The Fit-Criteria Assessment Plot (F-CAP) is an automated visual display of several model fit criteria recorded when fitting multi-GBTM for different and increasing numbers of clusters. For a closer description of this plot, readers are referred to the original publication[1]. As a gist, the F-CAP combines eight goodness-of-fit and model-adequacy criteria in compact graphs for several user stipulated varying k's (i.e., number of classes). The basic idea behind the F-CAP is that the user can see how indices change by increasing the number of latent trajectory clusters. The visual display of their behavior in such condensed form allows the assessment of different criteria side by side to reach a well-informed decision. In doing so, it fosters transparency in the process both for the user and the critical appraiser.

In our data, the F-CAP showed a clear elbow-kink for the information-based criteria: BIC, AIC and likelihood at k=5 - top plot. The APP, SD, mismatch and OCC criteria (second and third plots) were less discriminatory.

Once the final multi-GBTM model was selected, post hoc posterior probabilities of cluster-membership were estimated for each individual, who were then assigned to the cluster for which they had the highest posterior probability of assignment (PPA). As a rule of thumb[1], probabilities higher than 80% warrant the use of the assigned prevalence of the clusters in further analyses, without weighting the data with the probability of assignment. Because the final mean PPA to each latent cluster was over 0.8, we used a classify-analyse strategy for further analyses (i.e., clusters were taken as deterministic). For k>5 the sizes of the latent clusters become too small to be further considered (bottom plot). Altogether, the fit-criteria indicate that a 5-cluster solution is a good fit to the data.

1. Klijn, S. L., Weijenberg, M. P., Lemmens, P., van den Brandt, P. A., & Lima Passos, V. (2017). Introducing the fit-criteria assessment plot–A visualisation tool to assist class enumeration in group-based trajectory modelling. *Statistical methods in medical research*, **26(5)**, 2424-2436.
2. Heggeseth, B. C., Jewell, N. P. (2018). How Gaussian mixture models might miss detecting factors that impact growth patterns. *Annals of Applied Statistics*, **12(1)**, 222-245.

SAS syntax for the selected Model

PROC TRAJ DATA=traj.lasa_final_22 OUT=OF OUTEST=OE OUTPLOT=OP OUTSTAT=OS OUTPLOT2=op2 OUTSTAT2=os2 OUTPLOT3=op3 OUTSTAT3=os3 OUTPLOT4=op4 OUTSTAT4=os4 ;
ID id; VAR lim1-lim7; INDEP T1-T7; *disability outcome;
MODEL CNORM; MIN **0**; MAX **24**; NGROUPS **5**; ORDER **4 3 3 4 3**;
VAR2 wlog1-wlog7; INDEP2 T1-T7; *6m Walk outcome log transformed;
MODEL2 CNORM; MIN2 **1**; MAX2 **6**; NGROUPS **5**; ORDER2 **3 4 4 3 2**;
VAR3 grip1-grip7; INDEP3 T1-T7; *Grip outcome;
MODEL3 CNORM; MIN3 **0**; MAX3 **68**; NGROUPS **5**; ORDER3 **3 4 3 4 3**;
VAR4 gc1-gc7; INDEP4 T1-T7; *Centered values for grip;
MODEL4 CNORM; MIN4 -**31**; MAX4 **18**; NGROUPS **5**; ORDER4 **3 4 3 4 3**;
VAR5 wcl1-wcl7; INDEP5 T1-T7; *centered values for log 6m walk;
MODEL5 CNORM; MIN5 -**1.5**; MAX5 **2.5**; NGROUPS **5**; ORDER5 **3 4 4 2 3**;
MULTGROUPS **5**;
RUN;

eTable 2'a. Coefficients (estimate, error) constituting the trajectories of each measure, by trajectory cluster.

	6m Walk		Grip strength		Disability	
	Estimate	Error	Estimate	Error	Estimate	Error
Trajectory cluster 1						
Intercept	1.61	0.14	49.3	2.7	6.81	1.80
Linear	0.35	0.22	11.8	4.2	1.08	1.80
Quadratic	0.12	0.11	4.6	2.0	0.42	0.52
Cubic	0.02	0.02	0.8	0.4	0.03	0.04
Quartic	0.001	0.001	0.04	0.02	-	-
Trajectory cluster 2						
Intercept	1.40	0.12	29.6	1.1	4.46	1.45
Linear	0.78	0.19	6.0	1.2	1.33	1.46
Quadratic	0.33	0.09	1.6	0.4	0.83	0.42
Cubic	0.06	0.02	0.1	0.03	0.08	0.04
Quartic	0.003	0.001	-	-	-	-
Trajectory cluster 3						
Intercept	1.78	0.08	44.3	1.6	1.24	3.49
Linear	0.28	0.10	9.4	1.8	1.72	5.59
Quadratic	0.05	0.03	2.2	0.6	0.96	2.83
Cubic	0.006	0.003	0.2	0.1	0.21	0.56
Quartic	-	-	-	-	0.01	0.04
Trajectory cluster 4						
Intercept	2.06	0.06	36.6	2.5	3.47	2.62
Linear	0.08	0.07	23.5	4.1	1.99	4.19
Quadratic	0.02	0.02	10.1	2.1	0.58	2.10
Cubic	0.002	0.002	1.8	0.4	0.15	0.41
Quartic	-	-	0.1	0.03	0.01	0.03
Trajectory cluster 5						
Intercept	2.26	0.06	29.1	2.3	8.03	2.28
Linear	0.50	0.06	12.5	3.1	7.42	3.02
Quadratic	-	-	3.6	1.2	2.13	1.12
Cubic	-	-	0.3	0.1	0.20	0.12
Quartic	0.06	0.01	-	-	-	-

eTable 2a. Observed and predicted values for the 6m walk

	Wave 1: 1995-96	Wave 2: 1998-99	Wave 3: 2001-02	Wave 4: 2005-06	Wave 5: 2008-09	Wave 6: 2011-12	Wave 7: 2015-16
TC1: High-functioning							
Observed	6.3	7.0	7.2	7.2	8.0	8.4	10.3
Predicted	6.3	7.0	7.2	7.3	7.8	8.7	10.2
TC2: High-functioning, below-average grip strength							
Observed	6.7	7.8	7.7	7.8	8.9	9.3	10.2
Predicted	6.7	7.7	7.8	7.9	8.6	9.6	10.1
TC3: Functional decline							
Observed	7.5	9.0	9.9	11.2	13.9	18.2	23.7
Predicted	7.5	8.8	10.0	11.4	13.6	17.5	25.3
TC4: Functional decline, poor grip strength							
Observed	8.7	10.0	11.3	13.6	15.8	17.7	19.8
Predicted	8.7	9.9	11.5	13.5	15.7	17.9	19.8
TC5: Low-functioning							
Observed	14.6	21.8	21.8	27.0	25.6	23.9	-
Predicted	14.9	20.3	24.5	26.1	24.6	20.4	-

Note: TC = Trajectory cluster

eTable 2b. Observed and predicted values for grip strength

	Wave 1: 1995-96	Wave 2: 1998-99	Wave 3: 2001-02	Wave 4: 2005-06	Wave 5: 2008-09	Wave 6: 2011-12	Wave 7: 2015-16
TC1: High-functioning							
Observed	41.4	38.2	38.3	36.6	34.6	31.9	27.6
Predicted	41.3	38.5	37.7	36.9	34.8	31.5	27.8
TC2: High-functioning, below-average grip strength							
Observed	25.1	22.4	22.0	21.9	20.6	18.5	15.3
Predicted	25.0	22.7	21.8	21.5	20.8	18.9	14.9
TC3: Functional decline							
Observed	36.9	32.1	30.8	27.5	25.9	22.6	14.5
Predicted	36.8	32.5	30.0	28.3	26.0	22.0	15.0
TC4: Functional decline, poor grip strength							
Observed	21.5	17.5	17.5	17.7	15.4	12.9	11.8
Predicted	21.5	17.4	17.5	17.5	15.7	12.7	12.0
TC5: Low-functioning							
Observed	19.9	15.7	16.0	13.8	16.3	10.6	-
Predicted	19.9	16.0	15.2	15.5	14.8	11.0	-

Note: TC = Trajectory cluster

eTable 2c. Observed and predicted values for disability

	Wave 1: 1995-96	Wave 2: 1998-99	Wave 3: 2001-02	Wave 4: 2005-06	Wave 5: 2008-09	Wave 6: 2011-12	Wave 7: 2015-16
TC1: High-functioning							
Observed	0.8	1.2	2.4	4.3	5.2	7.2	9.1
Predicted	0.6	1.1	2.1	3.4	5.2	7.0	8.6
TC2: High-functioning, below-average grip strength							
Observed	1.3	2.1	3.1	5.7	6.5	9.1	8.8
Predicted	1.1	1.8	3.1	5.0	7.0	8.5	8.8
TC3: Functional decline							
Observed	4.8	7.7	10.1	13.7	14.8	15.5	16.2
Predicted	4.7	7.4	10.4	13.0	14.7	15.5	15.6
TC4: Functional decline, poor grip strength							
Observed	6.5	9.2	11.2	14.2	15.2	16.4	17.6
Predicted	6.4	9.0	11.5	13.6	15.1	16.1	17.0
TC5: Low-functioning							
Observed	13.5	16.2	15.9	16.8	17.0	18.1	-
Predicted	13.4	15.7	16.1	16.0	16.3	18.0	-

Note: TC = Trajectory cluster

Supplement 2: Additional tables

eTable 3a. Baseline characteristics associated with earlier attrition[a] during the study period.

	**Regression coefficient B**	**95% Confidence Interval**
Baseline age	0.11	0.10; 0.12
Sex	-0.07	-0.23; 0.10
Education in years (M, sd)	-0.01	-0.04; 0.01
Number of chronic diseases (M, sd)	0.17	0.11; 0.24
MMSE score (M, sd)	-0.13	-0.15; -0.10

[a] Based on the last wave of participation: partcipation up to an earlier wave represents earlier attrition

eTable 3b. Number of waves before drop-out[a] in the five trajectory clusters for each functional ability measure

	**Trajectory cluster number**					
	TC1<br>N=258	TC2<br>N=394	TC3<br>N=245	TC4<br>N=506	TC5<br>N=299	Total<br>N=1,702
6m Walk, M (sd)	4.1 (2.1)	3.8 (2.1)	3.1 (1.6)	2.7 (1.7)	1.6 (1.1)	3.0 (1.9)
Grip strength, M (sd)	4.0 (2.1)	3.7 (2.2)	3.1 (1.6)	2.6 (1.7)	1.7 (1.2)	3.0 (1.9)
Disability, M (sd)	4.2 (2.1)	4.0 (2.1)	3.3 (1.6)	2.9 (1.7)	2.1 (1.2)	3.2 (1.9)

[a] Participants are considered to have dropped out if they did not contribute data during >=3 consecutive waves and did not contribute data after that. Note, that a number of participants did not contribute data at a certain wave, did contribute data at one or more subsequent waves; they are considered to have contributed data up to the last wave at which they contributed.

eTable 4. Odd Ratios of socio-demographic characteristics and physical and cognitive health for each trajectory cluster, compared to the most favourable trajectory cluster. Multivariable, multinomial regression analysis

	Trajectory cluster's OR (95%CI)			
	**2 vs 1**	**3 vs 1**	**4 vs 1**	**5 vs 1**
Model 1 [a]				
Baseline age in quartiles				
- 70-74 vs 65-69	2.25 (1.38; 3.68)	2.39 (1.50; 3.79)	5.57 (3.22; 9.61) [cd]	10.63 (4.59; 24.62) [cd]
- 75-80 vs 65-69	4.88 (2.77; 8.61)	7.25 (4.31; 12.18)	28.60 (15.64; 52.29) [cd]	114.19 (49.03; 265.89) [cde]
- 81-88 vs 65-69	9.76 (4.71; 20.22)	9.54 (4.73; 19.25)	111.50 (53.25; 233.50) [cd]	786.43 (306.72; 2016.40) [cde]
Sex (female vs male)	82.60 (43.19; 158.00)	2.92 (1.38; 6.22) [c]	161.57 (82.07; 318.06) [d]	168.06 (82.63; 341.81) [cd]
Model 2 [b]				
Baseline age in quartiles				
- 70-74 vs 65-69	2.11 (1.27; 3.48)	2.26 (1.39; 3.70)	5.32 (3.01; 9.40)	10.03 (4.18; 24.07) [cd]
- 75-80 vs 65-69	4.46 (2.49; 8.00)	6.36 (3.66; 11.03)	23.64 (12.57; 44.46) [cd]	87.45 (36.17; 211.40) [cd]
- 81-88 vs 65-69	7.73 (3.62; 16.52)	9.34 (4.44; 19.65)	91.87 (42.10; 200.46) [cd]	620.61 (229.81; 1676.02) [cde]
Sex (female vs male)	82.74 (42.67; 160.45)	2.82 (1.30; 6.10) [c]	168.80 (83.57; 340.98) [cd]	181.90 (86.29; 383.45) [cd]
Education in years				
- 7-12 vs >=13	0.69 (0.93; 1.22)	1.19 (0.67; 2.11)	0.92 (0.49; 1.71)	1.02 (0.49; 2.11)
- <=6 vs >=13	1.04 (0.55; 1.99)	1.72 (0.89; 3.31)	1.84 (0.93; 3.66)	1.96 (0.90; 4.27)
Number of chronic diseases				
- 1 vs 0 diseases	1.09 (0.65; 1.82)	1.94 (1.09; 3.44) [c]	1.34 (0.75; 2.40)	3.15 (1.45; 6.82) [ce]
- 2 vs 0 diseases	1.39 (0.78; 2.48)	4.18 (2.27; 7.68) [c]	3.41 (1.84; 6.34) [c]	9.17 (4.15; 20.27) [ce]
- >=3 vs 0 diseases	1.31 (0.66; 2.64)	9.26 (4.74; 18.11) [c]	9.03 (4.49; 18.18) [c]	45.46 (19.40; 107.00) [cde]
MMSE score				
- scores 27-28 vs 29-30	1.13 (0.70; 1.83)	1.36 (0.85; 2.18)	1.21 (0.72; 2.03)	2.77 (1.47; 5.24) [cde]
- scores 25-26 vs 29-30	1.36 (0.73; 2.53)	1.94 (1.07; 3.49)	1.74 (0.92; 3.31)	4.98 (2.37; 10.46) [cde]
- scores <=24 vs 29-30	1.60 (0.78; 3.32)	1.12 (0.53; 2.37)	3.46 (1.69; 7.12) [cd]	8.77 (3.88; 19.82) [cde]

OR: Odds Ratio; CI: Confidence Interval
[a] Nagelkerke pseudo-Rsquare = 55.7%
[b] Nagelkerke pseudo-Rsquare = 63.8%
[c] OR differs significantly from OR(2 vs 1)
[d] OR differs significantly from OR(3 vs 1)
[e] OR differs significantly from OR(4 vs 1)

eTable 5. Comparison of models with baseline age and sex alone, with educational level alone, and with all three sociodemographics. Multivariable, multinomial regression analysis

	Trajectory cluster's OR (95%CI)			
	**2 vs 1**	**3 vs 1**	**4 vs 1**	**5 vs 1**
Model 1[a]				
Baseline age in quartiles				
- 70-74 vs 65-69	2.25 (1.38; 3.68)	2.39 (1.50; 3.79)	5.57 (3.22; 9.61)[cd]	10.63 (4.59; 24.62)[cd]
- 75-80 vs 65-69	4.88 (2.77; 8.61)	7.25 (4.31; 12.18)	28.60 (15.64; 52.29)[cd]	114.19 (49.03; 265.89)[cde]
- 81-88 vs 65-69	9.76 (4.71; 20.22)	9.54 (4.73; 19.25)	111.50 (53.25; 233.50)[cd]	786.43 (306.72; 2016.40)[cde]
Sex (female vs male)	82.60 (43.19; 158.00)	2.92 (1.38; 6.22)[c]	161.57 (82.07; 318.06)[d]	168.06 (82.63; 341.81)[cd]
Model 2[aa]				
Education in years				
- 7-12 vs >=13	1.14 (0.72; 1.79)	1.33 (0.80; 2.21)	1.20 (0.76; 1.91)	1.17 (0.68; 2.01)
- <=6 vs >=13	2.94 (1.77; 4.87)	2.12 (1.20; 3.76)	5.09 (3.09; 8.38)[cd]	5.65 (3.21; 9.97)[cd]
Model 3[ab]				
Baseline age in years				
- 70-74 vs 65-69	2.23 (1.36; 3.66)	2.36 (1.49; 3.76)	5.49 (3.17; 9.52)[cd]	10.58 (4.56; 24.60)[cd]
- 75-80 vs 65-69	4.92 (2.79; 8.69)	7.22 (4.29; 12.15)	28.77 (15.67; 52.81)[cd]	114.85 (49.15; 268.38)[cde]
- 81-88 vs 65-69	9.27 (4.46; 19.26)	9.38 (4.64; 19.00)	105.82 (50.30; 222.66)[cd]	756.47 (293.70; 1948.41)[cde]
Sex (female vs male)	78.39 (40.79; 150.54)	2.68 (1.25; 5.72)[c]	140.57 (71.07; 278.06)[d]	142.48 (69.68; 291.34)[d]
Education in years				
- 7-12 vs >=13	0.78 (0.45; 1.35)	1.35 (0.79; 2.33)[c]	1.04 (0.57; 1.88)	1.27 (0.64; 2.52)
- <=6 vs >=13	1.24 (0.67; 2.29)	1.99 (1.08; 3.66)	2.44 (1.28; 4.63)[c]	3.23 (1.57; 6.61)[c]

OR: Odds Ratio; CI: Confidence Interval
[a] Nagelkerke pseudo-Rsquare = 55.7%
[aa] Nagelkerke pseudo-Rsquare = 7.1%
[ab] Nagelkerke pseudo-Rsquare = 56.6%
[c] OR differs significantly from OR(2 vs 1)
[d] OR differs significantly from OR(3 vs 1)
[e] OR differs significantly from OR(4 vs 1)

eTable 6. Comparison of participants who fulfilled the stricter criteria of having provided data at a minimum of two waves for each outcome measure with participants who did not.

	**>=2 waves each measure (*N*=1083)**	**<2 waves any measure (*N*=619)**
Baseline age in years (M, sd)	74.0 (6.3)	78.9 (6.5)
- 65-69	82.5%	17.5%
- 70-74	73.2%	26.8%
- 75-80	59.4%	40.6%
- 81-88	39.4%	60.6%
Sex		
- Male	62.4%	37.6%
- Female	64.7%	35.3%
Education in years (M, sd)	9.0 (3.3)	8.4 (3.4)
- >=13	65.2%	34.8%
- 7-12	70.1%	29.9%
- <=6	56.7%	43.3%
Number of chronic diseases (M, sd)	1.6 (1.2)	2.0 (1.4)
- 0 diseases	67.2%	32.8%
- 1 disease	70.3%	29.7%
- 2 diseases	61.6%	38.4%
- >=3 diseases	55.9%	44.1%
MMSE score (M, sd)	27.3 (2.4)	25.1 (4.1)
- scores 29-30	79.0%	21.0%
- scores 27-28	69.6%	30.4%
- scores 25-26	57.9%	42.1%
- scores <=24	34.7%	65.3%

Note: Except for sex and education, the differences between all covariates are highly significant (F-value for age, chronic diseases, and MMSE (as continuous variables): 233.1, 30.2, and 205.4, respectively)
